# Formation, architecture, and persistence of oral biofilms: recent scientific discoveries and new strategies for their regulation

**DOI:** 10.3389/fmicb.2025.1602962

**Published:** 2025-07-09

**Authors:** Chengyuan Lv, Ziyi Wang, Zehui Li, Xialing Shi, Mingming Xiao, Yan Xu

**Affiliations:** ^1^Department of Surgical Oncology and General Surgery, First Hospital of China Medical University, Shenyang, China; ^2^Department of Thoracic Surgery, National Cancer Center/National Clinical Research Center for Cancer/Cancer Hospital, Chinese Academy of Medical Sciences and Peking Union Medical College, Beijing, China; ^3^Department of Thoracic Surgery, First Hospital of China Medical University, Shenyang, China; ^4^Department of Pathology, The People’s Hospital of Liaoning Province, Shenyang, China

**Keywords:** oral biofilm, oral disease, microbiome, colony, structure, persistence, bacterial adhesion, regulation

## Abstract

Complex microbial interactions occur on the surfaces within the oral cavity, where biofilms form highly organized ecosystems composed of diverse microbial communities and their metabolic products. These biofilms, typically located on the tooth surface or within the gingival sulcus, play a crucial role in both oral and systemic health. Recent studies have significantly improved our understanding of the mechanisms of biofilm formation, their structural characteristics, and their persistence over time. However, the intricate interactions between biofilms and the host, as well as their contributions to both local (e.g., dental caries and periodontitis) and systemic conditions, remain only partially understood. This mini-review summarizes recent scientific progress on the formation, structural dynamics, and ecological functions of oral biofilms. It also highlights emerging strategies for modulating biofilm composition and activity, the regulatory systems governing these interactions, and potential directions for microbiome-based therapies in future research.

## 1 Introduction

Oral biofilms are complex microbial consortia that adhere to both hard (e.g., teeth) and soft (e.g., gingiva) surfaces within the oral cavity, forming in response to mechanical forces, salivary flow, nutrient fluctuations, and tissue interfaces (Mirghani et al., 2022; Montelongo-Jauregui and Lopez-Ribot, 2018). Unlike biofilms in other parts of the body, oral biofilms are constantly influenced by mastication, intermittent dietary intake, and host immune factors, creating a highly dynamic microenvironment (Kolenbrander et al., 2010; Jakubovics and Kolenbrander, 2010). These biofilms play central roles in maintaining oral health but can also transition into pathogenic communities associated with caries, gingivitis, and periodontitis (Kinane et al., 2017; Murakami et al., 2018; Bowen et al., 2018; Larsen and Fiehn, 2017). More importantly, dysbiotic oral biofilms are increasingly implicated in systemic diseases, including cardiovascular disease, diabetes, Alzheimer’s disease, and chronic kidney disease, underscoring their broad clinical significance (Kurtzman et al., 2022; Yumoto et al., 2019).

The formation of oral biofilms is a multi-step process involving initial bacterial adhesion, following colonization, interbacterial communication, and maturation into a three-dimensional (3D) structured community (Mirghani et al., 2022; Marsh and Zaura, 2017). The extracellular polymeric substances (EPS)—comprising exopolysaccharides, proteins, lipids, and extracellular DNA (eDNA)—provide structural integrity and mediate metabolic interactions within biofilms (Karygianni et al., 2020; Cugini et al., 2019; Klein et al., 2015). Certain bacteria secrete EPS and surface adhesins to aggregate into polymers, which form a cohesive matrix that promotes attachment, protects against immune clearance, and limits antibiotic penetration. These features also complicate antimicrobial treatment, leading to persistent infections and therapeutic challenges (Mirghani et al., 2022; Kuboniwa and Lamont, 2010; Bjarnsholt et al., 2018; Zijnge et al., 2010; Crabbé et al., 2019).

In response to these challenges, recent research has focused on novel approaches to biofilm regulation, including natural products like plant-derived compounds, biosurfactants, probiotics, and nanomaterials. These agents target specific biofilm components or microbial communication systems (Benoit et al., 2019; Hu et al., 2019; Lee et al., 2020; Campbell et al., 2020). These strategies represent a shift from broad-spectrum antimicrobial eradication to targeted modulation aimed at restoring oral microbial balance.

This review provides a comprehensive and critical synthesis of recent findings on oral biofilm formation, structure, and persistence, along with emerging therapeutic strategies. By bridging structural biology, microbial ecology, and clinical innovation, this work aims to support the development of precise, microbiome-conscious interventions for managing biofilm-associated diseases.

## 2 Manuscript

### 2.1 Structural characteristics of oral biofilms

#### 2.1.1 Compositional components of oral biofilms

**Figure 1:**
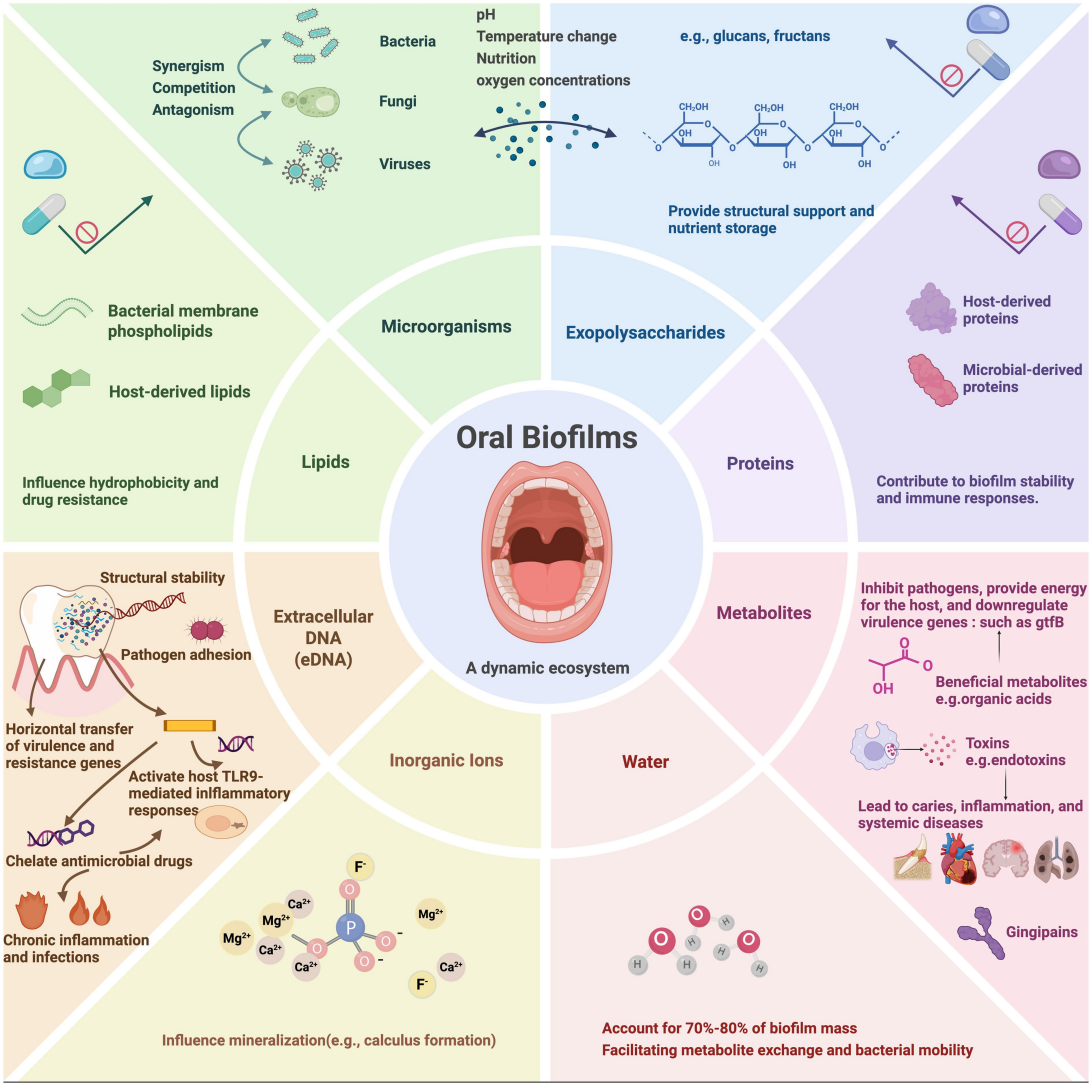
The components of oral biofilms. Oral biofilms are composed of eight elements (microorganisms, extracellular polymeric substances, proteins, metabolites, extracellular DNA, inorganic ions and water). The interactions of these elements contribute to formation and persistence of oral biofilms. Created with BioRender.com.

Oral biofilms comprise diverse microorganisms, with primary and secondary bacterial colonizers sequentially establishing the community structure (Kolenbrander et al., 2006; Marsh, 2006; Huang et al., 2011). These microorganisms secrete EPS contributes to the formation of biofilms. Oral biofilms are embedded in a highly organized EPS matrix, forming a highly organized matrix composed mainly of water, exopolysaccharides, proteins, lipids, inorganic ions, and extracellular DNA (eDNA). Exopolysaccharides, mainly produced by cariogenic bacteria such as *Streptococcus mutans*, serve as the structural backbone that stabilizes the biofilm and facilitates microbial adhesion and aggregation. Exoproteins contribute to structural integrity, enzymatic activity, and nutrient processing. Lipids are involved in biofilm hydrophobicity and barrier functions, while inorganic ions like calcium and magnesium facilitate cross-link matrix components and regulate mineralization. eDNA, originating from lysed cells, plays multifaceted roles in maintaining structural cohesion, promoting horizontal gene transfer, enhancing antimicrobial resistance through antibiotic chelation, and triggering host immune responses via TLR9 signaling pathways (Panlilio and Rice, 2021; Sharma and Rajpurohit, 2024; Kondo et al., 2022). Collectively, these components create a resilient and dynamic microenvironment that underpins the architecture and pathogenic potential of oral biofilms (Figure 1).

#### 2.1.2 3D architecture and functional properties of oral biofilms

Bacteria in the oral cavity form biofilms with complex 3D structures embedded within the EPS matrix (Zijnge et al., 2010). Confocal laser scanning microscopy has revealed that these biofilms comprise multiple layers, with different bacteria species occupying distinct ecological niches—enabling interspecies interactions and competition (Reichhardt and Parsek, 2019). Within this architecture, bacteria organize into diverse spatial arrangements, multilayered communities that give rise to microenvironments with varying nutrient gradients, oxygen levels, and metabolic activities. Early colonizers, such as *Streptococcus* spp., consume oxygen and reduce local oxygen tension, creating anaerobic niches that support obligate anaerobes, including pathogenic species implicated in periodontal disease. This ecological succession—from aerobic to anaerobic conditions—is thought to contribute to the maturation and pathogenic potential of oral biofilms. For instance, under aerobic conditions, the formation of *S. mutans* biofilms is inhibited, and alterations in surface glycosylation enzyme expression impact its virulence potential (Ahn et al., 2007). This architectural complexity confers mechanical stability and enhances resistance to antibiotics and immune defenses (Xiao et al., 2012). Spatial heterogeneity also influences bacterial gene expression and metabolism, promoting interspecies interactions and facilitating the shift from commensal to pathogenic communities characterized by increased virulence, immune evasion, and pro-inflammatory activity. As such, biofilm architecture represents a critical therapeutic target in managing biofilm-associated oral diseases (Zijnge et al., 2010; Bell et al., 2024).

#### 2.1.3 Bacterial interactions within oral biofilms

Bacterial interactions within biofilms involve synergistic, antagonistic, and neutral mechanisms, collectively influencing biofilm development, persistence, and pathogenicity (Marsh and Zaura, 2017; Maier, 2021). For example, *Porphyromonas gingivalis* and *Treponema denticola* mutually promote each other’s growth and virulence within periodontal biofilms, whereas *S. mutans* inhibit *Streptococcus sanguinis* via bacteriocins production— an example of niche competition, where one species suppresses another occupying a similar ecological niche (Ng et al., 2019; Kreth et al., 2005). Cooperative interactions can also promote antibiotic resistance through synergistic protective mechanisms. Beyond physical and metabolic interactions, quorum-sensing (QS) system plays a pivotal role in regulating collective bacterial behaviors. QS system is a cell-to-cell communication system in which bacteria detect population density via signaling molecules, thereby regulating biofilm formation, virulence expression, and resistance development. A well-known example is the use of *N*-acyl-homoserine lactones (AHLs) in Gram-negative bacteria to regulate biofilm maturation and pathogenic gene expression (Kumar et al., 2022; Asahi et al., 2010).

### 2.2 Process of oral biofilm formation

#### 2.2.1 Initial stage: initial adhesion and colonization

The initial stage of oral biofilm formation begins with pioneer species such as *S. mutans* adhering to the salivary pellicle, a unique oral structure composed of proteins (e.g., statherin, proline-rich proteins) and carbohydrates that selectively bind oral microorganisms. This attachment is usually mediated by adhesion factors, known as adhesins and biofilm-associated proteins such as *Streptococcus gordonii* SspB and *S. mutans* SpaP, which bacteria use to bind host-derived substances (Mirghani et al., 2022; Álvarez et al., 2022). Adhesion progresses from weak, reversible interactions (e.g., van der Waals forces) to stronger, irreversible binding as bacteria produce EPS, particularly glucans and fructans, which facilitate further colonization. Environmental factors such as pH, temperature, nutrient availability, and mechanical shear from salivary flow can also influence adhesion stability (Álvarez et al., 2022; Whittaker et al., 1996). Moreover, early colonizers can facilitate the recruitment of secondary species by exposing hidden receptors via sialidase and releasing signaling molecules such as competence-stimulating peptide (CSP) and autoinducer-2 (AI-2), thereby promoting increasingly community complexity (Jakubovics, 2015).

#### 2.2.2 Developmental stage: formation and diversity of bacterial communities

Following initial adhesion, biofilms undergo substantial structural and compositional changes as early colonizers proliferate and new microbial species are incorporated, increasing complexity and heterogeneity. Bacteria secrete EPS, which provide structural scaffold that facilitates interspecies interactions, including nutrient sharing and metabolic cross-feeding, thereby promoting microbial diversity (Cugini et al., 2019; Serrage et al., 2021; Koo et al., 2009). Pioneer colonizers like *Streptococcus* and *Actinomyces* contribute to this architecture by producing EPS and surface adhesions, enabling the attachment of secondary colonizers (Klein et al., 2015; Dige et al., 2009; de Oliveira et al., 2020). Environmental factors, including salivary composition and dietary habits, influence community development and diversity (Simon-Soro et al., 2022). For instance, short-term juice consumption has been reported to negatively affect the microbiota (Sardaro et al., 2025).

Traditional culture-based methods detect only a fraction of oral bacteria, many of which are non-culturable under standard conditions. Culture-independent techniques, such as 16S rRNA sequencing and metagenomics, have revealed a much broader microbial landscape, uncovering previously unknown phyla with potential roles in health and disease. This distinction between culturable and unculturable taxa highlights the complexity of oral microbiome and underscores the need for comprehensive analytical approaches.

As biofilms mature and become diverse, their intricate 3D architecture and protective EPS matrix confer increased resistance to antimicrobial agents and host immunity. Notably, microbial diversity itself has been associated with clinical outcomes—higher oral diversity correlates with significantly reduced all-cause mortality (Yu et al., 2024; Rudney et al., 2003). Interspecies interactions, including both cooperation and competition, further biofilms development and function. Certain probiotic strains, such as *Lactobacillus plantarum* and *Streptococcus salivarius* K12, play regulatory roles by modulating microbial composition and behavior. For example, *S. salivarius* K12, suppresses cariogenic biofilm formation by downregulating *S. mutans* glucosyltransferase genes (*gtfB*, *gtfC*, *gtfD*), thereby interfering with EPS synthesis and biofilm stability (Lee et al., 2021; Kim and Yoo, 2023; Ahn et al., 2018).

#### 2.2.3 Stable stage: stability and maintenance of the physiological state of oral biofilms

The stable stage marks the maturation of oral biofilms into structurally resilient, metabolically active communities capable of enduring environmental stress while supporting diverse microbial populations within spatially distinct microenvironments (Maier, 2021). This stability is maintained through synergistic interactions among key factors, including the EPS matrix, microbial metabolism, and intercellular signaling pathways (Cugini et al., 2019; Panlilio and Rice, 2021). The dense EPS framework serves as a physical barrier that impedes antibiotic penetration and promotes antimicrobial tolerance (Cugini et al., 2019; Panlilio and Rice, 2021). Concurrently, bacterial coordination—through QS and stress response systems—enhances structural integrity and collective resistance. A deeper understanding of the mechanisms governing oral biofilm stability and resilience is essential for developing targeted strategies to prevent and manage biofilm-associated oral diseases.

#### 2.2.4 Dispersion stage: biofilm dispersal and initiation of new colonization

The dispersion stage marks the final phase of the oral biofilm lifecycle, during which bacterial detach from mature biofilms and colonize new surfaces, initiating subsequent rounds of biofilm formation (Rumbaugh and Sauer, 2020). This stage is crucial for the propagation and persistence of microbial communities and plays a central role in the recurrence of oral infections.

Biofilm dispersion is triggered by various environmental and endogenous cues, including nutrient depletion, pH changes, accumulation of metabolic waste, and QS signals (Teschler et al., 2022). To facilitate release from the matrix, bacteria deploy mechanisms such as enzymatic degradation of EPS (e.g., glycoside hydrolases, DNases, and proteases), altered expression of adhesion molecules, and enhanced motility (Wang et al., 2023). Notably, dispersed cells often display increased virulence and antibiotic tolerance, posing significant challenges for therapeutic intervention.

As a biological transition between biofilm stability and renewed colonization, dispersion completes the biofilm lifecycle. Elucidating the regulatory mechanisms governing this process not only deepens our understanding of oral microbial ecology but also offers potential targets for disrupting biofilm persistence and transmission.

### 2.3 Persistence of oral biofilms

The mechanisms underlying biofilm formation—such as EPS production, spatial structuring, and interspecies communications—are not only essential for initial community establishment, but also play a pivotal role in the long-term persistence and resistance of oral biofilms. Understanding this continuum from formation to persistence is critical for identifying points of therapeutic vulnerability.

#### 2.3.1 Adaptability of oral biofilms to environmental changes

Oral biofilms exhibit remarkable adaptability to the ever-changing conditions of the oral cavity through metabolic reprogramming, gene regulation, and interspecies communication. *S.mutans*, a key cariogenic species, responds to oxidative stress via ActA-mediated acetylation of PykF, which reconfigures central carbon metabolism and enhances bacterial survival. In acidic environments, *S. mutans* upregulates proton pumps and aciduric enzymes to maintain intracellular pH homeostasis (Ma et al., 2024; Dashper and Reynolds, 1992; Sekiya et al., 2019). Conversely, *S. gordonii* produces hydrogen peroxide to suppress competitors while simultaneously enhancing its own antioxidant defenses (Zheng et al., 2011; Jakubovics et al., 2002).

Under nutrient-limited conditions, *S. mutans* modulates its phosphotransferase system to optimize carbohydrate uptake and activates gluconeogenesis to sustain energy production and promote exopolysaccharide synthesis (Vadeboncoeur and Pelletier, 1997; Zeng et al., 2022). Interspecies signaling further reinforces collective resilience: QS coordinates population-wide gene expression in response to the environmental stimuli, while cyclic dinucleotide signaling (e.g., c-di-AMP) regulates stress-response pathways and promotes biofilm stability (Peng et al., 2016; Gürsoy et al., 2017).

These adaptive mechanisms are interconnected—metabolic and transcriptional shifts support bacterial survival while simultaneously enhancing extracellular matrix production, which reinforces biofilm architecture and buffers against environmental stressors (Costa et al., 2023). Collectively, these mechanisms clarify how oral biofilms maintain persistence and structural integrity under environmental challenges.

#### 2.3.2 Interactions between oral biofilms and host immune system

Interactions between oral biofilms and the host immune system significantly affect oral health (Lang et al., 2010). Commensal bacteria within biofilms, such as *S. sanguinis,* a key health-associated colonizer, contribute to immune homeostasis by eliciting minimal pro-inflammatory responses and suppressing inflammation induced by periodontal pathogens (Nobbs and Kreth, 2019). In contrast, pathogenic species such as *P. gingivalis* and *F. nucleatum* can impair host immunity. *P. gingivalis* secretes gingipains that degrade a wide range of host proteins, including cytokines and complement proteins, thereby impairing immune surveillance. *F. nucleatum* releases outer membrane vesicles that activate pro-inflammatory signaling pathway, exacerbating immune responses (Hočevar et al., 2020; Chen et al., 2022). The extracellular matrix acts as a physical barrier, protecting embedded bacteria from immune cell attacks (Karygianni et al., 2020). Additionally, certain bacteria can evade immune detection; for example, *T. denticola* can alter its surface antigens and actively suppress host immune responses to avoid detection and clearance (Jo et al., 2014; Hajishengallis, 2014; Dashper et al., 2011). These intricate interactions, shaped by biofilm composition and host immune status, influence biofilm stability and persistence. A better understanding of these mechanisms is crucial for developing innovative therapies that selectively target pathogenic biofilms while preserving beneficial components of the oral microbiota.

#### 2.3.3 Antibiotic resistance mechanisms of biofilms

The antibiotic resistance of oral biofilms presents a significant challenge in the treatment of periodontal diseases and dental caries. Beyond serving as a structural scaffold, the EPS matrix functions as a physical and chemical barrier, limiting antibiotic diffusion and reducing antimicrobial efficacy (Cugini et al., 2019; Panlilio and Rice, 2021; Simon-Soro et al., 2022). For instance, the penetration of chlorhexidine into *S. mutans* biofilms is significantly reduced; even following apparent disinfection, residual biofilm structure facilitates secondary adhesion and reformation (Takenaka et al., 2016). Certain bacteria, such as *Streptococcus anginosus* and *Lactobacillus salivarius,* can reversibly enter a metabolically dormant state, enhancing survival under stress and reducing antibiotic susceptibility (Chávez de Paz et al., 2008; Suppiger et al., 2020). Furthermore, in response to antimicrobial exposure, bacteria within biofilms may also undergo phenotypic shifts, including the upregulation of efflux pumps that actively expel antibiotics and reduce intracellular concentrations (Alav et al., 2018; Sionov and Steinberg, 2022; Cieplik et al., 2019). Moreover, intercellular communications, particularly via QS system, plays a crucial role in resistance. *S. mutans,* for instance, activates the CSP-ComDE system to promote the formation of multidrug-resistant persister cells, thereby enhancing biofilm persistence and resistance (Leung et al., 2015).

### 2.4 Oral biofilms in health and diseases

#### 2.4.1 Association between oral biofilms and oral health

Biofilms play a dual role in oral health. They are not inherently pathogenic but function as dynamic ecological communities essential for maintaining oral homeostasis. When dominated by commensal bacteria in healthy individuals, oral biofilms act as a barrier against external pathogens and stimuli, thereby contributing to a stable oral ecosystem. In this state, they modulate host immune responses, promote immune tolerance, and prevent excessive inflammation. This beneficial relationship reflects the concept of oral eubiosis, wherein balanced microbial interactions support tissue integrity and resist microbial dysbiosis (Santacroce et al., 2023; Zanetta et al., 2025). Beneficial species such as *Weissella cibaria* and members of genus *Lactobacillus* contribute to oral health by producing antimicrobial compounds, downregulating virulence genes such as *gtf* B, and preventing the adhesion of pathogens like *S. mutans* (Zanetta et al., 2025; Kang et al., 2023; Giordani et al., 2021). However, under certain conditions, this balanced environment may shift. When pathogenic bacteria become dominant, biofilms can transition into a dysbiotic state, promoting oral diseases, contributing to systemic disorders, and fostering antimicrobial resistance (Kurtzman et al., 2022; Yumoto et al., 2019).

#### 2.4.2 Association between oral biofilms and systemic diseases

Numerous studies have demonstrated a strong association between oral health, particularly the oral microenvironment, and overall systemic health. Bacteria involved in oral diseases can translocate into the bloodstream, triggering systemic inflammation and contributing to the onset of various related conditions (Kurtzman et al., 2022). For instance, Xiong et al. confirmed in animal models that gingipains produced by *P. gingivalis* induce insulin resistance by proteolytically degrading insulin receptors, promoting diabetes development (Liu et al., 2024). *P. gingivalis* has also been implicated in the pathogenesis of Alzheimer’s disease by inducing tau degradation (leading to impaired microtubule stability and neuronal dysfunction) and promoting Aβ_1–42_ deposition (a key component of amyloid plaques) via gingipains, thereby driving neurodegeneration (Dominy et al., 2019; Loughman et al., 2023).

The spirochete *T. denticola* has been shown to contribute directly to the progression of cardiovascular diseases, such as atherosclerosis (Chukkapalli et al., 2014). Additionally, bacteria present in oral biofilms including *Streptococcus pneumoniae*, *Prevotella* spp., and *Veillonella* spp. can reach the lower respiratory tract through microaspiration or inhalation, especially in elderly or immunocompromised individuals, thereby increasing the risk of pneumonia and chronic obstructive pulmonary disease (Pu et al., 2020; Mammen et al., 2020; Imai et al., 2021). Furthermore, *T. denticola*, *Tannerella forsythia*, and *Prevotella intermedia* have been shown association with chronic kidney disease by inducing the release of pro-inflammatory factors (e.g., IL-1β, TNF-α, IL-6, IL-17), which exacerbate renal inflammation, immune dysregulation, and renal endothelial damage (Li et al., 2021; Pontes and Chikte, 2020).

### 2.5 Emerging strategies for oral diseases based on oral biofilms

The emergence of antibiotic resistance in oral diseases has prompted the development of alternative treatment strategies. Recent studies have highlighted various promising approaches—including natural products, probiotics, biosurfactants, nanomaterials, enzymes, and biomolecules—that primarily function through three key mechanisms: direct anti-biofilm activity, biological immunomodulation, and oral microbiome balance regulation. These strategies offer comprehensive solutions for biofilm-associated oral diseases.

#### 2.5.1 Direct anti-biofilm activity

Antibiofilm strategies have become an active area of research, with diverse agents—such as antimicrobial peptides, natural compounds, biosurfactants, and nanoparticles—targeting different stages of biofilm development or specific components like EPS and extracellular nucleic acids. Recent studies have identified extracellular RNA (eRNA) as a key component of eDNA networks within biofilms formed by pathogens such as *Pseudomonas aeruginosa,* streptococci, and *Staphylococcus aureus* (Lee et al., 2020; Chiba et al., 2022; Mugunthan et al., 2023). Although eRNA is generally unstable and its structural role remains incompletely understood, emerging evidence suggests that it may serve as a promising target for disrupting oral biofilm formation (Mugunthan et al., 2023). For example, *Aronia melanocarpa* extracts significantly inhibit initial biofilm development by degrading eRNA in oral streptococcal biofilms (Lee et al., 2020).

Traditional Chinese medicine also exhibits anti-biofilm activity. For example, *Paeoniae Radix Alba* exhibits inhibitory effects against *S. mutans* biofilms, with albiflorin identified as its active constituent (Liu et al., 2025). Additionally, natural products such as stem extracts of *Rhamnus prinoides* (gesho) have been found to prevent biofilm formation in co-culture of *S. mutans* and *Candida albicans* (Campbell et al., 2020). New biosurfactants like rhamnolipids have demonstrated efficacy against biofilms formed by pathogenic bacteria such as *Aggregatibacter actinomycetemcomitans* Y4 (Yamasaki et al., 2020). Furthermore, nanoparticle-mediated treatments are emerging as innovative approaches, capable of generating of reactive oxygen species under acidic conditions to degrade biofilm matrix and eliminate pathogens like *S. mutans* (Benoit et al., 2019; Hu et al., 2019).

#### 2.5.2 Biological immunomodulation

Biological immunomodulation represents another emerging therapeutic strategy for oral diseases, functioning through multiple mechanisms to inhibit biofilm formation and restore oral homeostasis. *Weissella cibaria* has been shown to effectively suppress biofilm formation by various species, likely through competitive inhibition and downregulation of pro-inflammatory pathways (Kang et al., 2023). Similarly, probiotic bacteria such as *Lactobacillus* and *Bifidobacterium* spp. contribute to oral health by enhancing the production of anti-inflammatory cytokines (Mahdizade Ari et al., 2024). Beyond probiotics, certain immunomodulatory agents, such as D-galactose, have demonstrated potential in preventing pathogenic bacterial biofilms while promoting the growth of commensal streptococci (Ryu et al., 2020). Incorporating immunomodulators into oral healthcare strategies holds significant promise for the prevention and management of biofilm-associated oral diseases.

#### 2.5.3 Oral microbiome balance regulation

Oral biofilms are dynamic ecosystems that can exert both beneficial and detrimental effects; therefore, complete eradication is neither feasible nor desirable. Instead, modulating the oral microbiome toward a more health-promoting state is a promising strategy. For example, probiotics, like *Lactobacillus* species, can effectively suppress inflammation-associated pathogenic biofilms while promoting oral microbial balance (Ng et al., 2019). Although excessive level of short-chain fatty acids (SCFAs)—metabolic byproducts of the oral microbiota—are usually associated with oral dysbiosis, growing evidence highlights their beneficial roles. SCFAs can inhibit pathogens like *S. gordonii* by disrupting biofilm formation and suppressing virulence expression, and they also help alleviate chronic inflammation and contribute to a more balanced microbiome (Park et al., 2021; Leonov et al., 2023).

**Figure 2:**
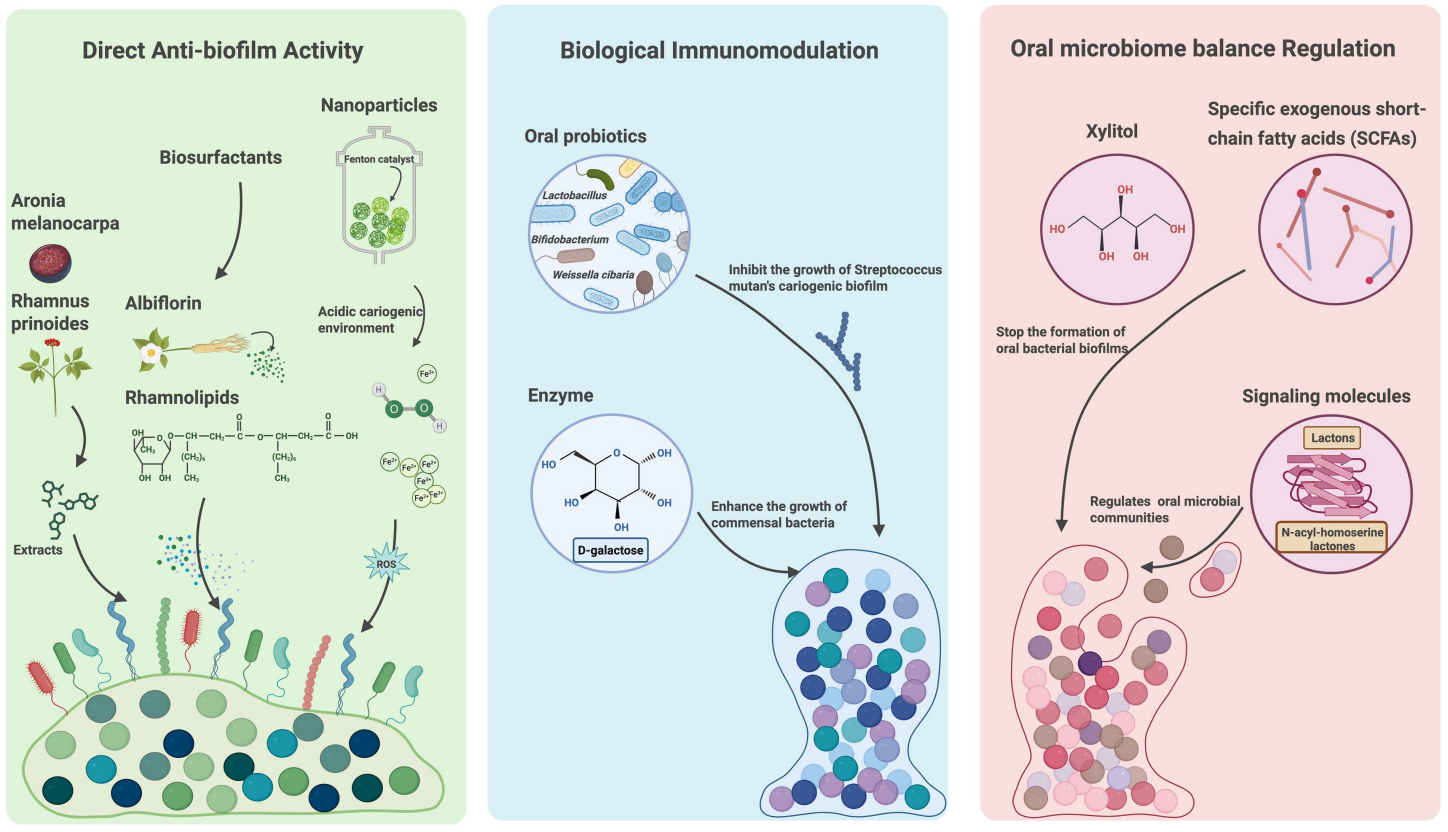
Emerging strategies for oral diseases based on oral biofilms. Emerging treatment strategies targeting oral biofilms are classified into direct anti-biofilm activity, biological immunomodulation, oral microbiome balance regulation based on the mechanisms. The treatment approaches include natural products, probiotics, biosurfactants, nanomaterials, enzymes and biomolecules. Created with BioRender.com.

Natural compounds such as xylitol, *Aronia melanocarpa*, and *Rhamnus prinoides* have also demonstrated the potential to support beneficial bacterial growth and enhance the oral microbiome homeostasis (Lee et al., 2020; Campbell et al., 2020; Teixeira Essenfelder et al., 2019). Furthermore, targeted interference with biofilm signaling molecules, such as AHLs in QS system, offers a means of selectively modulating microbial composition and suppressing pathogenic biofilm formation (Ahn et al., 2018; Parga et al., 2023). Collectively, these approaches facilitate oral microbial re-equilibrium and provide innovative strategies for the prevention and long-term management of biofilm-associated oral diseases (Figure 2).

### 2.6 Future direction of treatment strategies

In recent years, emerging therapeutic approaches increasingly emphasize selective targeting of pathogenic biofilms while preserving beneficial commensals—a paradigm shift from traditional broad-spectrum antimicrobials, which often disrupt oral microbial homeostasis and potentially leading to secondary infections or the emergence of resistant strains.

To address this, novel approaches have been proposed. For example, certain natural compounds and antimicrobial peptides can specifically inhibit pathogenic bacteria by recognizing distinct surface structures or metabolic features or downregulating the expression of *gtf* genes in *S. mutans*, thereby disrupting its extracellular polysaccharide synthesis and biofilm formation without affecting non-cariogenic streptococci (Xu et al., 2012). Similarly, probiotics such as *S. salivarius* K12 exhibit selective suppression of *S. mutans* colonization through competitive exclusion and modulating local microenvironment, indirectly supporting the growth of beneficial species (Begić et al., 2023). Additionally, QS-based interventions further enable disruption of pathogenic signaling networks without exerting bactericidal pressure, offering a potential route to disarm pathogenic biofilms while maintaining microbial homeostasis. These strategies emphasize regulation over eradication, aiming to restore a balanced and health-associated oral microbiome.

## 3 Conclusion

Oral biofilms, complex assemblages of microorganisms and their byproducts, play a pivotal role in both oral and systemic health. The formation and maturation of oral biofilms is a dynamic, multistage process, resulting in structurally intricate ecosystems that enable microbial survival, interaction, and adaptation. Consequently, oral biofilms can exert either protective or pathogenic effects. Their persistence and resistance to the environmental stressors have long challenged researchers and clinicians. Traditional strategies aimed at non-selective eradication are increasingly regarded as suboptimal. In contrast, emerging approaches emphasize modulation—shifting the composition and activity of biofilms toward a beneficial, health-promoting state. Advances in microbial and molecular technologies have deepened our understanding of oral microbial ecology, paving the way for more precise and sustainable interventions. As research in this field accelerates, growing attention is paid to the dualistic roles of biofilms in maintaining homeostasis and promoting disease. Future efforts, guided by multidisciplinary insights, should prioritize the targeted regulation of biofilms to foster eubiosis, enabling effective prevention and management of oral diseases while preserving microbial balance.
